# Glaciated valleys in Europe and western Asia

**DOI:** 10.1080/17445647.2014.921647

**Published:** 2014-05-23

**Authors:** Günther Prasicek, Jan-Christoph Otto, David R. Montgomery, Lothar Schrott

**Affiliations:** ^a^ Department of Geoinformatics – Z_GIS, University of Salzburg, Salzburg, Austria; ^b^ Department of Geography and Geology, University of Salzburg, Salzburg, Austria; ^c^ Department of Earth and Space Sciences, University of Washington, Seattle, USA; ^d^ Department of Geography, University of Bonn, Bonn, Germany

**Keywords:** geomorphology, geomorphometry, automation, semantics, glaciation, Europe

## Abstract

In recent years, remote sensing, morphometric analysis, and other computational concepts and tools have invigorated the field of geomorphological mapping. Automated interpretation of digital terrain data based on impartial rules holds substantial promise for large dataset processing and objective landscape classification. However, the geomorphological realm presents tremendous complexity and challenges in the translation of qualitative descriptions into geomorphometric semantics. Here, the simple, conventional distinction of V-shaped fluvial and U-shaped glacial valleys was analyzed quantitatively using multi-scale curvature and a novel morphometric variable termed Difference of Minimum Curvature (DMC). We used this automated terrain analysis approach to produce a raster map at a scale of 1:6,000,000 showing the distribution of glaciated valleys across Europe and western Asia. The data set has a cell size of 3 arc seconds and consists of more than 40 billion grid cells. Glaciated U-shaped valleys commonly associated with erosion by warm-based glaciers are abundant in the alpine regions of mid Europe and western Asia but also occur at the margins of mountain ice sheets in Scandinavia. The high-level correspondence with field mapping and the fully transferable semantics validate this approach for automated analysis of yet unexplored terrain around the globe and qualify for potential applications on other planetary bodies like Mars.

## 1. Introduction

The extent and chronology of past glaciations in Europe have been studied by geoscientists since the nineteenth century. Agassiz (1840) recognized erratic boulders and glacial striations as indicators for a once-extensive European ice shield, Penck and Brückner (1909) reconstructed ice ages from moraines and terraces to produce large-scale maps, and van Husen (1987) mapped the last glacial maximum (LGM) extent of the eastern European Alps. Recently, Ehlers and Gibbard (2004a, 2004b) and Ehlers, Gibbard, and Hughes (2011) reviewed the record of Quaternary glaciations around the world, providing a collection of digital data sets of glacial extent from various sources. For the vast majority of the documentation of glacial extent, mapping was carried out in the field. Only over the last several decades has supporting information been extracted from satellite-based remote-sensing data (e.g. Andrews, Davis, & Wright, 1976; Boulton & Clark, 1990), digital terrain models (e.g. Berthier *et al*., 2007; Mohr, Reeh, & Madsen, 1998), or a combination of both (e.g. Clark, 1997; Paul, Kääb, Maisch, Kellenberger, & Haeberli, 2002).

Since the advent of GIS-assisted mapping in the 1990s, a large variety of tools have been developed to extract geometric properties from digital elevation models to support geomorphological mapping (Seijmonsbergen, Hengl, & Anders, 2011). Going one step further, recent (semi-) automated mapping concepts not only supply a human mapper with geometric terrain analysis, but account for the subsequent geomorphological interpretation as well (MacMillan & Shary, 2009). To do so, quantitative classification rules are needed. In geomorphology, landform description is largely based on qualitative aspects, rather than on quantified and measurable attributes. Translation of such descriptions into morphometric semantics poses a considerable challenge and few landform classification studies have modeled semantics explicitly (Eisank, Drăguţ, & Blaschke, 2011).

(Semi-) automated geomorphological mapping promises two substantial advantages over conventional methods: time-efficient analysis of large areas, and objective landscape classification. In this study, we tried to achieve both, based on simple morphometric semantics. We automatically mapped areas dominated by glacially incised valleys in Europe and western Asia using an algorithm introduced by Prasicek, Otto, Montgomery, and Schrott (2014). The crucial semantics are based on the conventional wisdom of V-shaped fluvial and U-shaped glacial valley cross sections. The development of glacial valleys featuring a distinctive U-shape during the Pleistocene can be expected mostly in temperate alpine environments, exhibiting considerable relief and affected by basal glacial erosion (Benn & Evans 2010; Sugden, 1974). This qualifies our algorithm for the identification of areas affected by warm-based alpine glaciation. Northern parts of Europe were buried under mainly cold-based continental ice (Ehlers & Gibbard, 2004a; Hättestrand & Stroeven, 2002), whereas the rest of Europe experienced warm-based glaciers in alpine regions (Ehlers & Gibbard, 2004a; Hubbard & Sharp, 1995). Nevertheless, a complex spatiotemporal pattern of continental glaciations and partially warm-based mountain ice sheets led to the development of U-shaped valleys in northern Europe as well. Here, deep glacial troughs at the margins of mountain ice sheets are accompanied by high-elevation, low-relief surfaces and relic landscapes indicating a bimodal form of glacial erosion (Kleman, Stroeven, & Lundqvist, 2008; Steer, Huismans, Valla, Gac, & Herman, 2012; Sugden, 1978). To validate our results, we compare the predicted distribution of glaciated valleys with field mapping of the LGM ice extent in mountainous areas not covered by continental glaciation. We exclude regions where distinctive glacial valleys did not develop like extensive outlet glaciers and minor glaciations of summit regions.

A raster map of areas with valleys incised by warm-based alpine glaciation is the result of this work. The map covers major parts of Europe, as well as Armenia, Azerbaijan, Georgia, Turkey and fractions of Iran, Iraq and Syria. This extension of the study area across European Borders allows analysis of the Caucasus and adjacent mountain ranges. The input data are hydrologically conditioned terrain models (HydroSHEDs; http://hydrosheds.cr.usgs.gov) with a resolution of 3 arc seconds (80 m), derived from the Shuttle Radar Topography Mission (SRTM) of the United States Geological Survey (USGS). Therefore, terrain north of the 60th parallel is not included in this survey. Besides our automated mapping results, elevation from the SRTM data and field mapping of glacial extent during the LGM are displayed on the map for validation. The field mapped data are part of a global dataset, compiled by Ehlers and Gibbard (2004a, 2004b). Due to the scale of our investigation, their overview dataset is included on the map, instead of the detailed LGM extents collected from numerous separate projects. The LGM of the Tatra Mountains was compiled from other sources (Kotarba, 1992; Lindner, Dzierżek, Marciniak, & Nitychoruk, 2010). Manual mapping of the extent of the LGM in Iran and south-eastern parts of Turkey were excluded, because of uncertainties in the data. The map covers an area of about 1 × 10^6^ km^2^ and has a WGS 84 / World Mercator projection (EPSG 3395).

## 2. Methods

We automatically mapped glaciated valleys across Europe and western Asia based on cross-sectional valley shape. We assumed that valleys with a prevailing fluvial imprint typically reveal a V-shaped cross-section with uniformly steep slopes, whereas glacial valleys tend to have a U-shaped profile with a changing slope gradient. Distinction between these two genetic types of valley transects can be performed by grid-based analysis of multi-scale curvature to depict valley shape (Prasicek *et al*., 2014). Accurate calculation of the required land surface parameters (LSPs) such as curvature and upstream drainage area poses an additional challenge due to the large study area and the sphericity of the Earth.

### 2.1. Curvature differences between glacial and fluvial valleys

Curvature indicates to what extent an n-dimensional object is curved. In geomorphometry, curvature calculated from digital terrain models (DTMs) can highlight divergent and convergent parts of the landscape and has important implications for surface processes (Carson & Kirkby, 1972). The most widely used methods for curvature calculation on regular grids are those of Evans (1979) and Zevenbergen and Thorne (1987). To derive curvature on an irregular digital surface, a polynomial model is fitted to a 3 × 3 neighborhood and centered on a cell in the grid. In addition, a plane intersecting the fitted polynomial model has to be specified because surface curvature varies with orientation. Curvature can then be calculated from the intersection graph. Wood (1996) adapted the approach of Evans (1979) to perform computation of LSPs on varying neighborhoods for multi-scale analysis and implemented it in LandSerf (http://landserf.org). Curvature is calculated as the change of slope in radians per 100 m. To account for differences in object size, the cumulative change of slope is given – a dimensionless ratio providing similar values for similar shapes independent of scale. Prasicek *et al*. (2014) used this approach to perform multi-scale curvature calculation in a three-dimensional environment for automated identification of glaciated valleys. They used minimum curvature representing the most concave transect on a surface.

**Figure 1.**
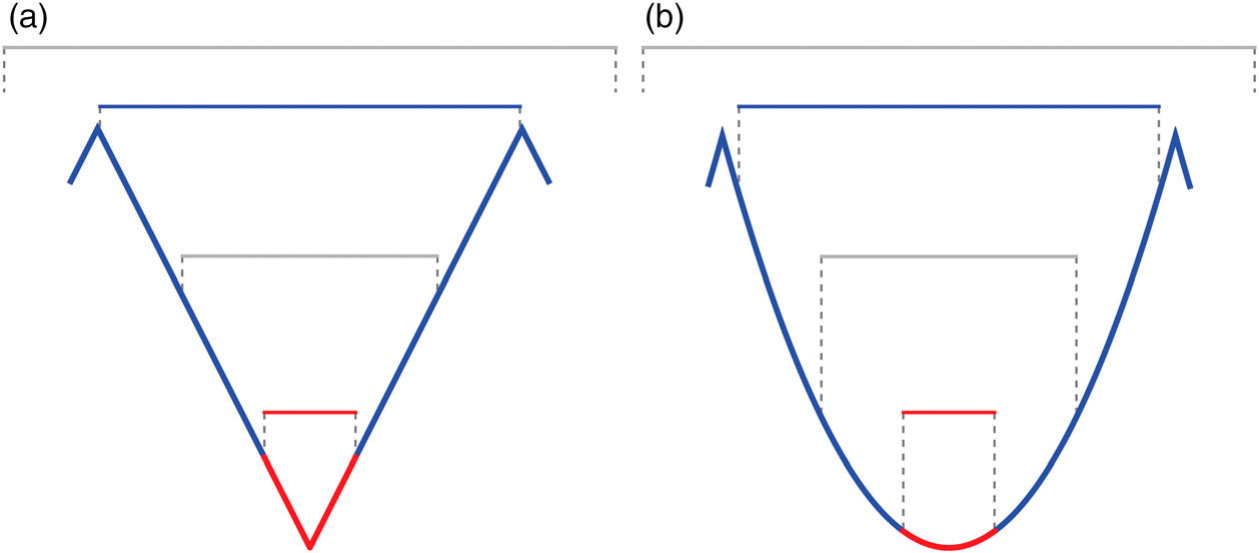
Two-dimensional scheme illustrating differences in cross-sectional morphology of idealized, similar sized V-shaped (a) and U-shaped (b) valleys (bold blue) and thalweg subsections (bold red). Different scales of multi-scale curvature analysis are represented by horizontal bars. The V-shaped graph has the same shape at all scales. Instead, the U-shaped transect reveals a different form if varying amounts of the graph are analyzed. Curvature calculated and compared over multiple scales can be employed to quantify these variations and identify glaciated valleys.

The theoretical principles of the methodology are illustrated in a two-dimensional example in Figure 1. Cross-sectional profiles of artificial V-shaped fluvial (Figure 1(a)) and U-shaped glacial (Figure 1(b)) valleys of similar extent show different shapes depending on the scale of investigation (horizontal bars). The fluvial valley cross section has the same shape for all scales. Instead, the glacial valley transect reveals a different form if varying amounts of the graph are analyzed. To distinguish between V-shaped and U-shaped cross sections, minimum curvature values depicting the change of slope along the most concave transect, the valley cross section, can be compared for two different scales: first, a reference scale that mainly depicts the center of the cross section (Figure 1, red bars); and second, a scale that covers the whole valley width (Figure 1, blue bars). For the V-shaped profile, the curvature values are similar for the red and the blue scale of investigation, 3.59 and 3.71, respectively (Figure 1(a)). In contrast, the curvature increases with analysis scale from 0.74 (red) to 3.71 (blue) for the U-shaped graph (Figure 1(b)). For an automated analysis of large data sets, variations in valley width have to be considered. While the reference scale can be a fixed extent, the second scale of investigation varies with valley width and has to be adjusted automatically. For this, we employed the concept of characteristic scale (Wood, 2009): an LSP is calculated over multiple scales and the extent where it becomes most extreme is specified. For U-shaped valleys, curvature will be most concave when calculated for the whole valley cross section. For V-shaped valleys, concavity will be similar for all scales. If the scale of investigation is large enough to overlap with major ridges (Figure 1, light gray bars), curvature takes less extreme values and such scales will automatically not be taken into account. Therefore, this approach automatically adjusts to valley width. Finally, curvature is calculated for a variety of scales and the two results are compared. The reference scale (Figure 1, red lines and red bars), which is the smallest scale and identical for valleys of any size, is used to measure the form of the central parts of a cross section. In contrast, the maximum concavity scale is automatically defined by the scale at which the largest value of concavity is measured, and therefore inherently varies depending upon valley width (Figure 1, blue lines and blue bars).

On a DTM, the scale for curvature calculation is represented by the width of the moving window (e.g. 3 × 3 cells), and only cells forming the drainage line of a valley (thalweg, valley bottom in Figure 1) carry information about cross-sectional valley shape relevant for our approach. Therefore, cells constituting ridges and hillslopes are ruled out by a drainage area cutoff, here set to 1 km^2^ following findings on the onset of convergent terrain by other workers (Brardinoni & Hassan, 2006; Ijjasz-Vasquez & Bras, 1995; McNamara, Ziegler, Wood, & Vogler, 2006; Montgomery & Foufoula-Georgiou, 1993). For all thalweg cells Minimum curvature is calculated for a variety of scales ranging from 3 × 3 to 79 × 79 cells (240 to 6300 m). From this, the Difference of Minimum Curvature (DMC) is calculated by subtracting the reference-scale curvature (3 × 3 cells) from maximum concavity scale curvature (variable scale). For a detailed description of the applied methodology, please refer to Prasicek *et al*. (2014).

**Figure 2.**
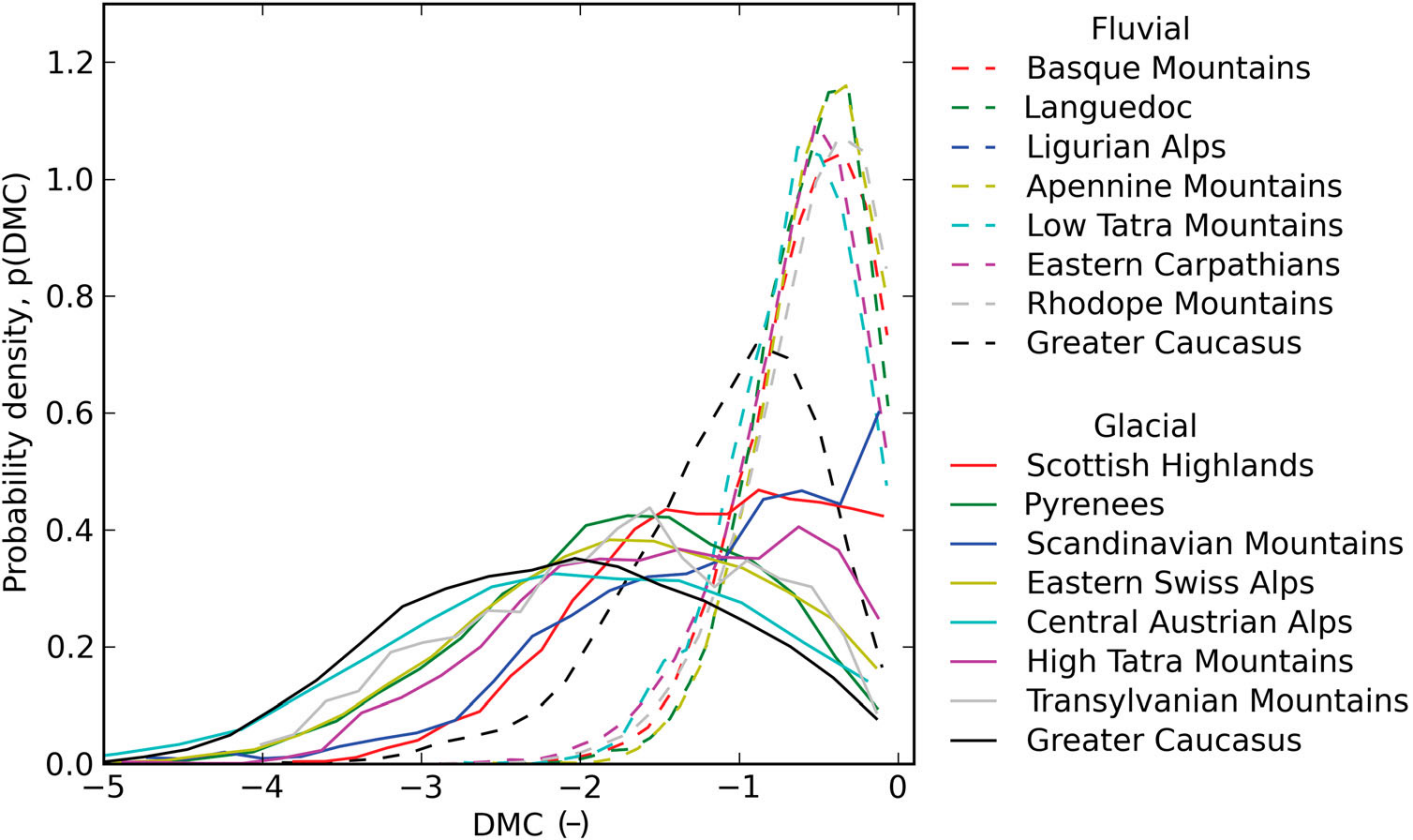
Probability density functions of Difference of Minimum Curvature (DMC) for 8 fluvial and 8 glacial sample sites. A threshold to differentiate glacial from fluvial valleys is suggested at a DMC of approximately −1.15, where most of the fluvial sample site graphs intersect with the glacial ones.

Finally, to identify glacial valleys in an automated manner, an empirical DMC threshold has to be determined. Prasicek *et al*. (2014) suggested that a single DMC threshold may exist which can be used to identify glaciated valleys around the globe. We tested this hypothesis for Europe and western Asia by defining 8 fluvial and 8 glacial sample areas and plotting their DMC probability density functions to derive and compare their DMC thresholds (Figure 2, see Section 3 for details). The sample sites are displayed on the Main Map. Sample sites had to be clearly dominated by well-developed fluvial or glacial valleys, avoiding terrain that appeared flat relative to the applied moving window sizes, to adequately depict the specific cross-sectional shape of these valley types.

Generalization plays a major role in visualizing our results on a map. It is needed because: (i) only thalweg cells, a limited part of the DTM that cannot be displayed on a small-scale map, are used for classification; (ii) as the theoretical framework is based on idealized valley cross sections, single cells may not be representative due to the heterogeneity of valley morphology; and (iii) the surrounding terrain most likely belongs to the same class as the thalweg cells. For this, we classify all thalweg cells by applying our DMC threshold and then determine the class (either *glacial* or *non-glacial*) holding the majority of thalweg cells for quadrangles of 180 × 180 cells (14.4 × 14.4 km, approximately 200 km^2^). The quadrangles have to meet two criteria: they have to be large enough to hold a representative number of thalweg cells (no empty quadrangles); and they should be small enough to take into account small areas like single massifs where distinctive glacial valleys have developed.

### 2.2. LSP calculation for large areas

Land surface parameters depict geometric attributes of a digital terrain model. If calculated for an extensive study area, projection issues have to be considered. Depending on the LSP, different types of projections are most suitable. Local LSPs are calculated for a limited neighborhood on a grid. For this work, calculation of minimum curvature required grid cells to truly cover equal distances in every direction throughout the study area. Owing to the sphericity of the Earth, no single map projection is capable of this over very large areas. Therefore, we split the original HydroSHEDS grid covering the whole study area and projected the tiles to the corresponding UTM zones (WGS 84, 29N-39N). We then calculated multi-scale curvature for these tiles.

In addition, upstream drainage area had to be calculated per cell to apply the drainage area cutoff. This regional LSP is computed in two steps: calculation of flow direction, specified by the steepest gradient in a 3 × 3 neighborhood (D8 algorithm introduced by Jenson and Domingue (1988)); and calculation of flow accumulation, representing the number of grid cells (or area) draining into each cell of the grid. Projecting the DTM between those two steps is not possible, because flow direction cannot be interpolated. Therefore, it has to be calculated in one projection for the whole study area. To do so, we chose a conformal Mercator projection (EPSG 3395), ensuring that local angles (3 × 3 kernels) are reproduced correctly. Another advantage of the Mercator projection is its simplicity, allowing the weighting of per-cell area for calculation of flow accumulation. The same procedure can be carried out on a geographic coordinate system, however, this would bias the flow directions, dependent on latitude. The length of a parallel depends on latitude which is not considered in evaluations based on geographic coordinate systems hence affects the calculation of slope and aspect. Consequently, topographic gradients appear lower towards east or west, whereas cells tend to be exposed towards these directions. However, this issue can be expected to only affect the flow direction of a limited number of cells located directly on watersheds.

## 3. Results & discussion

The DMC threshold required to distinguish between fluvially and glacially incised valleys was derived empirically from eight fluvial and eight glacial sample sites. The results are shown in Figure 2. In general, probabilities switch for all samples at a DMC of approximately −1.15 and we applied this value to the entire study area to predict the existence of glaciated valleys. Only the glacial Norwegian fjords sample and the fluvial Greater Caucasus sample differ from the other areas. This can be explained by the related morphology: The Caucasus sample shows a large amount of sediment fill in fluvial valleys, and the Norwegian fjords sample, which is known to represent glaciated U-shaped terrain (e.g. Steer *et al*., 2012), does not show glacial valleys as distinctive as the other samples due to missing cell values on sea-covered valley floors. Unfortunately, global bathymetry data available from the General Bathymetric Chart of the Oceans (GEBCO) has a maximum resolution of 30 arc seconds (GEBCO_08) which is too low for our purposes. A workaround for the fjords would give grid cells representing the sea a value of zero instead of ‘no data’, which would produce a flat valley floor and classify the fjords as *glacial*. However, this would lead to similar results for fluvial valleys. Hence our preference was to use ‘no data’ cell values and consistently interpret the sea surface as ‘terrain of unknown morphology’.

The general switch of probabilities for the 16 fluvial and glacial samples from all over Europe and western Asia at a DMC of −1.15 supports the hypothesis of a globally uniform DMC threshold, posited by Prasicek *et al*. (2014). However, our threshold differs significantly from those applied by Prasicek *et al*. (2014) in North America: −0.55, −0.70, and −0.71, for the Sawtooth Mountains, the Sierra Nevada, and the Olympic Mountains, respectively. This is probably a consequence of different resolution data sources. Prasicek *et al*. (2014) used the National Elevation Dataset (NED) of the United States with a resolution of 1 arc second, whereas this work was based on HydroSHEDS data with a cell size of 3 arc seconds. This led to slightly different geometries and demanded for a larger drainage area cutoff (1 km^2^ instead of 0.1 km^2^), which consequently affected curvature values. Nevertheless, DMC thresholds for various sample sites with the same drainage area cutoff and calculated from datasets with the same resolution seem to be similar.

The automatically identified areas with glaciated valleys are displayed on the Main Map, complemented by the manually mapped LGM extent from Ehlers and Gibbard (2004a, 2004b). In mountainous areas carved by warm-based alpine glaciations, the distribution of glaciated valleys conforms to the manually mapped LGM extent. The three large mountain ranges affected by extensive alpine glaciations during the Pleistocene, the Pyrenees, the Alps, and the Greater Caucasus, are clearly predicted by the automated mapping algorithm. Consistently, extensive piedmont glaciers that lack glacial valley incision and mountain ranges dominated by fluvially incised valleys are not identified. In northern Europe, covered by Pleistocene continental glaciation, U-shaped valleys detected by our algorithm are limited to uplands in Great Britain and Ireland, and to the Scandinavian Mountains. In western Scandinavia, the distribution of U-shaped valleys conforms to the extent of partially warm-based Pleistocene mountain ice sheets reported by Kleman *et al*. (2008).

While the big picture is depicted accurately, some finer-scale misclassification indicates the limits of the applied algorithm. Fluvial valleys with a wide sediment-filled valley floor or deep canyons can be mistaken for glacial imprint. However, this mostly applies to single flow lines which are generalized to *non-glacial* during the regionalization step, according to the majority of cells in their neighborhood. Nevertheless, clustering of these valleys occurs in limited areas leading to misclassification of isolated quadrangles. For further discussion of general classification issues refer to Prasicek *et al*. (2014).

**Figure 3.**
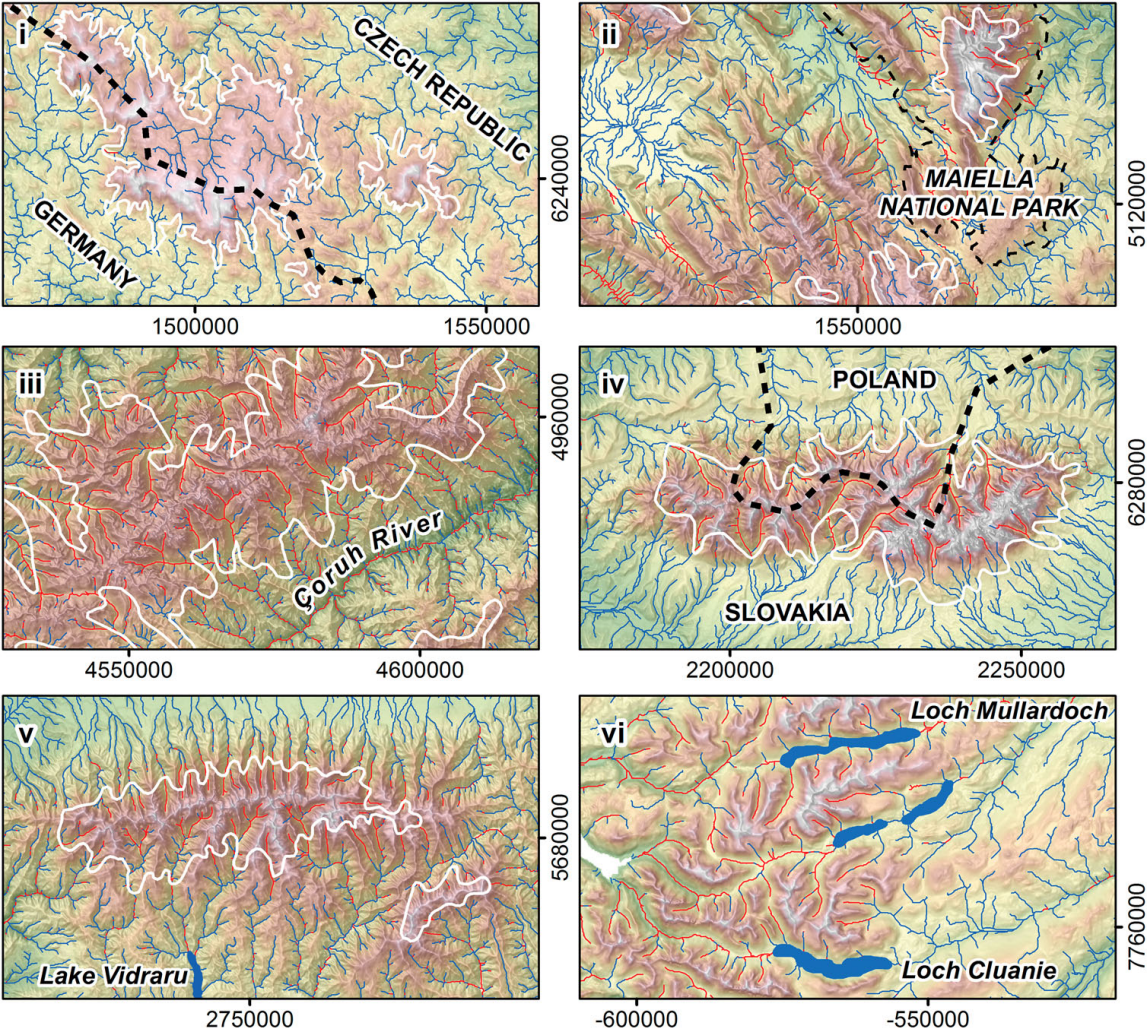
Comparison of the field mapped LGM extent (white outline) and the original thalweg classification derived from our algorithm (red: glacial, blue: non-glacial). (i) Bohemian Forest, Germany/Czech Republic; (ii) Apennine Mountains, Italy; (iii) Pontic Mountains, Turkey; (iv) Tatra Mountains, Slovakia/Poland; (v) Transylvanian Mountains, Romania; (vi) Scottish Highlands, UK (entirely covered by former continental glaciation). Spatial reference: WGS84 / World Mercator (EPSG 3395).

 Figure 3 and insets on the Main Map illustrate six examples for different classification issues from this project. Here, instead of the regionalized data, we present the originally interpreted thalwegs, classified as either *glacial* or *non-glacial*. The Bohemian Forest (i) and numerous other regions with small-scale LGM-glaciations do not show development of morphologically distinctive glacial valleys. Therefore, these glaciations are not predicted by the automated mapping algorithm. In the Apennine Mountains (ii), as well as in the Cantabrian Mountains, the southern Balkan Mountains, and the eastern Pontic Mountains, isolated fluvial quadrangles are misclassified as glacial due to deep fluvial canyons with a lack of V-shape. Glaciation in eastern Turkey, mostly situated in the Pontic Mountains (iii) and in the Taurus Mountains, was quite extensive and glacial valleys were identified by our methodology. Nevertheless, the western part of the Pontic Mountains was not classified *glacial*, because Pleistocene ice cover was limited to summit regions and distinctive glacial valleys did not develop. Conversely, the deeply incised canyon of the Çoruh River in the eastern Pontic Mountains leads to a number of cells misclassified as *glacial*. In the Taurus Mountains, our results match the field mapping only partially, due to glaciated plateaus lacking distinctive valley development and adjacent deeply incised canyons. In the Tatra Mountains (iv) and the Transylvanian Mountains (v), as well as in the central Balkan Mountains, the Rhodope Mountains, and on Corsica, glacial valleys coincide with former local glaciations. In northern Europe, glacial valleys mostly occur in the Highlands of Scotland (vi), Wales, and Ireland, and in the Scandinavian Mountains.

## 4. Conclusion

We present a map of morphometrically classified areas with glacial valleys in Europe and western Asia. The methods applied for automated mapping are based on simple assumptions on the differences in cross-sectional shape between glacially and fluvially incised valleys and accordingly derived morphometric semantics. The validated results illustrate the advantages and the drawbacks of automated geomorphological mapping. Objective landscape classification and large dataset processing allow automated morphometric analysis at a continental scale and processing of about 10^6^ km^2^ in a short time. As hypothesized previously (Prasicek *et al*., 2014), a single empirically defined threshold could be applied throughout the study area to objectively identify glaciated valleys. Although ambiguity in morphological features leads to finer-scale misclassification, the ‘big picture’ is depicted accurately and the presented small-scale map constitutes an overview of the spatial distribution of U-shaped valleys carved by warm-based alpine glaciation in Europe and western Asia automatically derived from digital terrain data.

Automated mapping of U-shaped valleys, a conventional indicator for past glaciation, is a simple and novel example for the application of morphometric semantics and may be applied to identify terrain features with U-shaped or V-shaped cross sections in a regional manner on less well-explored terrain around the globe and on other planetary bodies like Mars, where evidence for glacial remains is extensively investigated yet controversial (Head, Mustard, Kreslavsky, Milliken, & Marchant, 2003). Further research will focus on the combined application of multiple indicators, the detection of more subtle changes in cross-sectional valley morphology, and on the calculation of a ‘degree of glaciatedness’. In general, analysis of multi-scale curvature offers a promising avenue for the definition of geomorphometric semantics.

## Software

The geographic information system LandSerf (Wood, 1996) and particularly the LandScript Editor were used to calculate multi-scale curvature. Processing and analysis of grid tiles and sample areas was carried out based on Python and NumPy arrays. The final map was compiled in ArcGIS.

Main Map: Glaciated Valleys in Europe and Western Asia
