# Supplementary material for: Glaciated valleys in Europe and western Asia
Source: J Maps. 2014 May 23;11(2):361–70. doi: 10.1080/17445647.2014.921647 (PMC4786831; doi:10.1080/17445647.2014.921647)
Supplement: Main Map: Glaciated Valleys in Europe and Western Asia [file tjom_a_921647_sm5370.pdf]

Glaciated valleys in Europe and western Asia

Günther Prasicek, Jan-Christoph Otto, David R. Montgomery, and Lothar Schrott

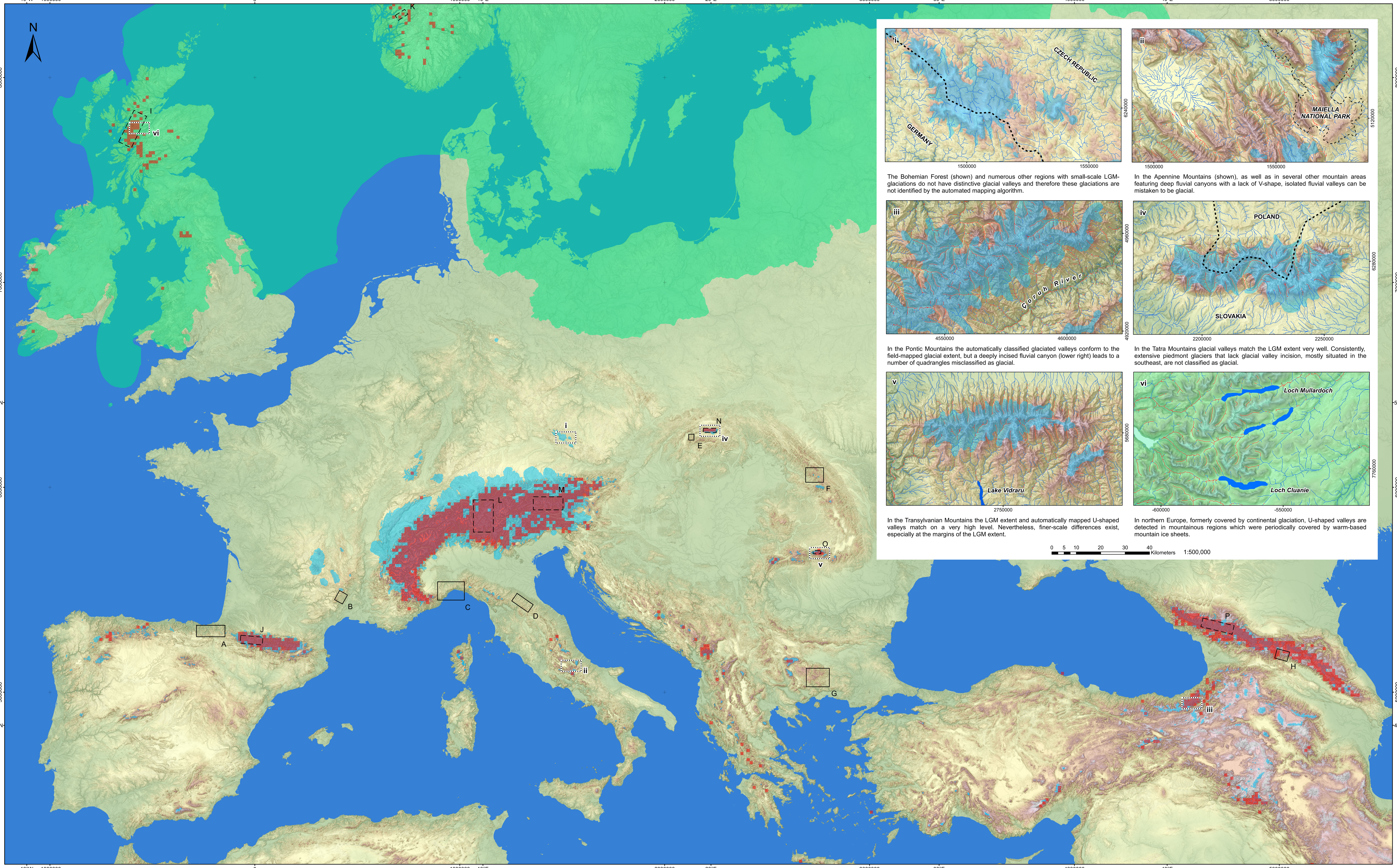

i: The Bohemian Forest (shown) and numerous other regions with small-scale LGM-glaciations do not have distinctive glacial valleys and therefore these glaciations are not identified by the automated mapping algorithm.

ii: In the Apennine Mountains (shown), as well as in several other mountain areas featuring deep fluvial canyons with a lack of V-shape, isolated fluvial valleys can be mistaken to be glacial.

iii: In the Pontic Mountains the automatically classified glaciated valleys conform to the field-mapped glacial extent, but a deeply incised fluvial canyon (lower right) leads to a number of quadrangles misclassified as glacial.

iv: In the Tatra Mountains glacial valleys match the LGM extent very well. Consistently, extensive piedmont glaciers that lack glacial valley incision, mostly situated in the southeast, are not classified as glacial.

v: In the Transylvanian Mountains the LGM extent and automatically mapped U-shaped valleys match on a very high level. Nevertheless, finer-scale differences exist, especially at the margins of the LGM extent.

vi: In northern Europe, formerly covered by continental glaciation, U-shaped valleys are detected in mountainous regions which were periodically covered by warm-based mountain ice sheets.

0 5 10 20 30 40 Kilometers 1:500,000

Field-mapped extent of the last glacial maximum (LGM), continental glaciation

(Ehlers, J., & Gibbard, P. L. (Eds.). (2004). Quaternary Glaciations - Extent and Chronology: Part I (Vol. 1) & Part III (Vol. 3). Amsterdam: Elsevier)

Field-mapped LGM extent, alpine glaciation

(Ehlers, J., & Gibbard, P. L. (Eds.). (2004). Quaternary Glaciations - Extent and Chronology: Part I (Vol. 1) & Part III (Vol. 3). Amsterdam: Elsevier)

Automatically mapped areas with glaciated valleys

Terrain covered by inset maps

Fluvial sample areas (A-H)

Glacial sample areas (I-P)

Elevation (m): 5500 – -30

Automatically classified thalwegs (inset maps only)

Glacial thalweg

Fluvial thalweg

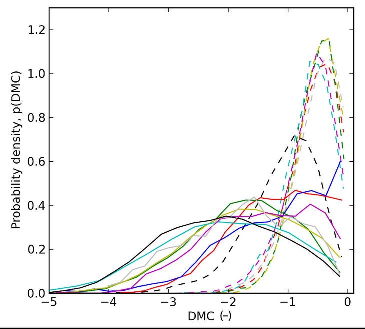

Fluvial sample areas	Glacial sample areas
A) Basque Mountains	I) Scottish Highlands
B) Languedoc	J) Pyrenees
C) Ligurian Alps	K) Scandinavian Mountains
D) Apennine Mountains	L) Eastern Swiss Alps
E) Low Tatra Mountains	M) Central Austrian Alps
F) Eastern Carpathians	N) High Tatra Mountains
G) Rhodope Mountains	O) Transylvanian Mountains
H) Greater Caucasus	P) Greater Caucasus

0 125 250 500 750 1,000 Kilometers

1:6,000,000

Base map (elevation and hill shade) compiled from hydrologically conditioned SRTM tiles (HydroSHEDS).
World Mercator conformal projection (EPSG 3395)
1984 World Geodetic System (wgs84)

© Journal of Maps, 2014

The map shows automatically detected quadrangles with glacially incised valleys using hydrologically conditioned digital terrain models (HydroSHEDS). The distinction between glacial and fluvial valleys is based on the conventional interpretation of U-shaped glacial and V-shaped fluvial valley cross sections. U-shaped glacial troughs are commonly associated with strong basal erosion and therefore this method is qualified to identify areas which experienced warm-based alpine glaciations. A simple morphometric variable termed Difference of Minimum Curvature (DMC) was calculated for thalweg cells and holds information about variations in valley cross-sectional curvature. Subsequently a DMC threshold was derived from probability density plots for 8 glacial and 8 fluvial sample areas (A – P). At a DMC of approximately -1.15 the probabilities for being either fluvial or glacial switch for most of these samples (see Figure on the left). This single DMC threshold was applied to the entire study area with promising results indicating a fully transferable methodology.

In regions coined by warm-based glacial erosion, glaciated valleys are a good predictor of glacial extent. However, deviations occur if distinctive glaciated valleys did not develop, for example in areas formerly covered by large piedmont glaciers at the northern fringe of the Alps, or in case of Pleistocene glaciations limited to summit regions. In northern Europe, formerly covered by continental glaciation, glacial valleys developed as well due to the periodic presence of partially warm-based mountain ice sheets. However, fjords are not identified due to lacking data in sea-covered terrain.

Finer-scale misclassification can occur in deep canyons and due to abundant sediment fill in fluvial valleys. In this case, single non-glacial quadrangles maybe mistaken for glacial terrain. The Inlet maps illustrate more detailed examples for classification issues and relations between LGM extent and glacial valleys.
